# 24780 Investigating the role of mycobacterial lipid antigens and CD1-restricted T cells in host-protective tuberculosis immunity using a guinea pig model

**DOI:** 10.1017/cts.2021.692

**Published:** 2021-03-30

**Authors:** Macallister Harris, James Dilisio, Hadley Gary, Edward Chan, D. Branch Moody, Brendan Podell

**Affiliations:** 1 Colorado State University; 2 University of Colorado of Denver; 3 University of Colorado Anschutz Medical Campus; 4 Harvard Medical School

## Abstract

ABSTRACT IMPACT: Examining lipid immunity for Mycobacterium tuberculosis in a translatable Guinea pig model may serve as a critical foundation for the creation of an efficacious human lipid based vaccine against tuberculosis. OBJECTIVES/GOALS: CD1 is a group of glycoproteins on antigen-presenting cells (APCs) that present lipid antigens to T cells. Mycobacterium tuberculosis (Mtb) has a lipid-rich cell wall which is essential for the pathogenesis of tuberculosis. Our goal is to determine the frequency, phenotypes, and functionality of CD1 T cells against Mtb using the guinea pig model. METHODS/STUDY POPULATION: Guinea pigs serve as the best translational model for CD1 immunology as they have both group 1 and group 2 CD1 complexes, comparable to human CD1. We performed ex-vivo and in-vivo experiments to analyze lipid antigen-specific CD1 T cell responses with Mtb infection. Assays to detect lipid-specific CD1 T cell activation include cellular proliferation, cytotoxicity assays, and interferon-gamma (IFNγ) release assay (Elispot) using both synthetic and Mtb-derived lipids. We isolated and characterized CD1 T cells using tetramerized CD1 complexes loaded with specific Mtb lipids. Spatial interaction between lipid loaded CD1 APCs with CD1 T cells were demonstrated by immunohistochemistry (IHC). Lastly, we will investigate the impact of lipid-based immunology via knockdown and overexpression of CD1 complexes. RESULTS/ANTICIPATED RESULTS: The cytotoxicity assay demonstrated that the CD1b1 and CD1b3 complexes play roles in the presentation of Mtb lipids, specifically glucose monomycolate, and mycolic acid, as noted by T cell killing of fibroblasts that express specific CD1 complexes that can present Mtb lipids. Similarly, cellular proliferation exhibited lipid specific T cell proliferation. IFNγ production by the stimulated CD1-restricted T cells (Elispot) was weak indicating CD1 T cells may not produce IFNγ. IHC successfully showed CD1 APCs in lungs and spleens of infected guinea pigs. It is anticipated that knocking out CD1 expression will lead to impaired immunity, and increase severity of disease as noted by pathologic lesions/bacterial burden, and systemic spread; in contrast, CD1 enhancement will limit the severity of tuberculosis. DISCUSSION/SIGNIFICANCE OF FINDINGS: We characterized CD1 T cells in infected guinea pigs at the tissue level, demonstrating Mtb lipid immunology. As a result, we laid the groundwork for investigating whether augmenting lipid immunity in the guinea pig model will enhance immunity against tuberculosis. Fruition of such work may lead to the development of effective tuberculosis vaccines.

